# Efficacy and toxicity of KRAS^G12C^ inhibitors in advanced solid tumors: a meta-analysis

**DOI:** 10.1186/s12957-024-03449-8

**Published:** 2024-07-16

**Authors:** Shoutao Dang, Shuyang Zhang, Jingyang Zhao, Wei Li

**Affiliations:** Cancer Center, Beijing Tongren Hospital, Capital Medical University, No. 2, Xihuan South Road, Yizhuang Town, Beijing, China

**Keywords:** KRAS^G12C^ inhibitors, Solid tumors, Meta-analysis

## Abstract

**Background:**

The efficacy and toxicity of KRAS^G12C^ inhibitors were evaluated for advanced solid tumors in several studies; however, the results were not fully consistent.

**Methods:**

Clinical trials evaluating KRAS^G12C^ inhibitors for advanced solid tumors were searched from PubMed, Embase, and Cochrane Library online databases up to 31st December 2023. The characteristics of the studies and the results of objective response rate (ORR), disease control rate (DCR), duration of response (DoR), progression-free survival (PFS) rate, overall survival (OS) rate, and treatment-related adverse events (trAEs) were extracted.

**Results:**

Ten studies with 925 heavily pretreated advanced patients harboring KRAS^G12C^ mutation were included. For total population, the pooled analysis of ORR was 28.6% (95%CI, 21.2-36.6%), DCR was 85.5% (95%CI, 82.2-88.6%), PFS rate at 6 months (PFS6) was 49.6% (95%CI, 41.4-57.9%), PFS rate at 12 months (PFS12) was 26.7% (95%CI, 19.8-34.1%), OS rates at 6 months (OS6) was 76.2% (95%CI, 68.8-82.9%), OS rates at 12 months (OS12) was 47.8% (95%CI, 38.6-57.0%). The pooled analysis of any grade trAEs was 79.3% (95%CI, 66.2-90.0%) and grade three or more trAEs was 24.4% (95%CI, 16.7-32.9%). The median time to response and DoR results from individual data were 1.39 months (95%CI, 1.37–1.41 months) and 10.54 months (95%CI, 7.72–13.36 months). Sotorasib had significantly lower pooled incidences of any trAEs (OR, 0.07, 95%CI, 0.03–0.14) and grade three or more trAES (OR, 0.34, 95%CI, 0.24–0.49) compared with adagrasib.

**Conclusions:**

KRAS^G12C^ inhibitors have good ORR, DCR, PFS rate, OS rate, tolerable trAEs, and early response with long duration in advanced solid tumors; however, most of the pooled results were heterogeneous. Sotorasib has shown better safety results.

**Supplementary Information:**

The online version contains supplementary material available at 10.1186/s12957-024-03449-8.

## Background

Kirsten rat sarcoma (KRAS) gene is one of the most frequently mutated oncogenes, and it regulates cell signaling through downstream mitogen activated protein kinase (MAPK) and phosphatidylinositol 3-kinase (PI3K) pathways by encoding its protein [1]. KRAS mutations can cause active state of tyrosine kinase and constant activation of downstream signaling which lead to cell proliferation, migration, and tumorigenesis [2–4]. Despite KRAS mutations may play an important role in tumorigenesis, there has been no meaningful progress in targeted therapy against these mutations [5]. KRAS^G12C^ mutation (glycine-to-cysteine substitution at codon 12) is one of the most common KRAS mutations, and it may occur in a variety of tumor types, such as non-small-cell lung cancer (NSCLC), colorectal cancers (CRC), pancreatic cancers, and other cancers [6].

In recent years, a new binding site on the KRAS^G12C^ protein was found, and several small molecule drugs targeting the KRAS^G12C^ mutation have been developed [7–9]. Sotorasib, the first KRAS^G12C^ inhibitor, can irreversibly and specifically inhibit the KRAS^G12C^ protein, prevent downstream signaling, and thereby hinder oncogenesis [10]. In 2020, a phase 1 trial of sotorasib in patients with advanced solid tumors with KRAS^G12C^ mutation suggested promising anticancer activity and tolerable toxicity in heavily pretreated patients [11]. Subsequent phase 2 and 3 clinical trials for NSCLC, CRC, and pancreatic cancers also indicated encouraging results [12–15]. Other KRAS^G12C^ inhibitors such as adagrasib, garsorasib, and divarasib were also investigated in several clinical trials [16–20]. The phase 1 KRYSTAL-1 study suggested that adagrasib was well tolerated and effective for advanced solid tumor patients with KRAS^G12C^ mutation. Another phase 1 study evaluating divarasib for advanced solid tumor patients with KRAS^G12C^ mutation showed durable clinical responses with mostly low-grade adverse events. A more recent phase 1 study evaluating garsorasib for NSCLC patients with KRAS^G12C^ mutation also presented encouraging antitumor activity and good safety profiles. However, the results of KRAS^G12C^ inhibitors were not fully consistent.

We searched clinical trials that evaluating KRAS^G12C^ inhibitors for advanced solid tumors from online databases. And we conducted a meta-analysis to evaluate the efficacy and toxicity of KRAS^G12C^ inhibitors in advanced solid tumors with KRAS^G12C^ mutation.

## Methods

### Data sources and search strategy

Studies were searched via PubMed, Embase, and Cochrane Library database up to 31st December 2023. We used the Medical Subject Headings (MeSH) terms and their entry terms as follows: “KRAS G12C OR sotorasib OR lumakras OR AMG-510 OR AMG 510 OR AMG510 OR adagrasib OR MRTX849 OR MRTX-849 OR ARS853 OR ARS-1620 OR garsorasib OR BI-2852”.

### Inclusion and exclusion criteria

This meta-analysis followed the PRISMA (Preferred Reporting Items for Systematic Reviews and Meta-analyses) statement [21].

The inclusion criteria were as follows: (a) patients with KRAS^G12C^ mutation advanced solid tumors; (b) there was at least one group that was treated with KRAS^G12C^ inhibitors monotherapy; (c) there was at least one outcome of objective response rate (ORR), disease control rate (DCR), duration of response (DoR), progression-free survival (PFS), overall survival (OS), treatment-related adverse events (trAEs) was reported.

The exclusion criteria were as follows: (a) patients were treated with KRAS^G12C^ inhibitors plus other systemic therapy; (b) the data could not be extracted; (c) the study was not published in English; (d) retrospective study, conference abstracts, case reports, comments, reviews, animal studies, and mechanistic studies.

### Quality assessment and data extraction

The risk of bias was assessed by two investigators (Shoutao Dang and Shuyang Zhang) according to the Methodological Index for Nonrandomized Studies (MINORS) tool [22].

The data were extracted by the same two investigators. The following information were included: first author, publication year, trial phase, sample size, eligible patients, interventions and control group, and outcomes of the ORR, DCR, DoR, PFS, OS, and trAEs. The PFS rates, OS rates, and individual DoR data were indirectly extracted from survival curves and swimmer plot figures. Any disagreement was discussed for consensus.

### Statistical analysis

This meta-analysis was performed by Stata 17. The pooled results of ORR, DCR, PFS rate, OS rate, and incidences of trAEs were performed by “metaprop” statistical program. The subgroup pooled results were performed by “subgroup by” statistical program. The heterogeneity of the studies was estimated by using the I^2^ statistics. Random-effects model was used for all results. Kaplan-Meier estimates for extracted individual data of DoR were produced by “graph Kaplan-Meier survivor function”. Log-rank test was calculated by “sts test” statistical program to compare among different subgroups of DoR. The odds ratio (OR) was calculated by “csi” statistical program to compare the pooled incidences between different subgroups. Statistical significance was set at *p* < 0.05.

## Results

### Study and data selection

**Fig. 1:**
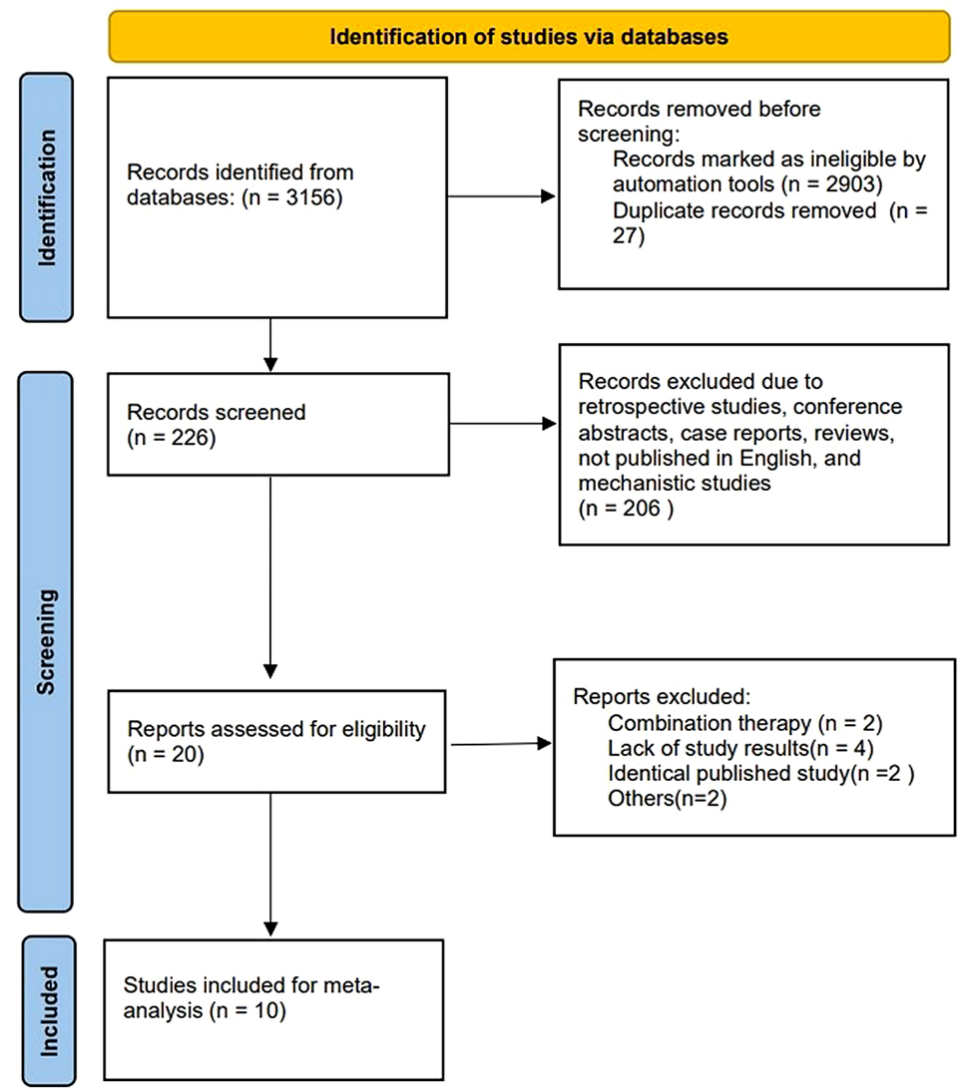
Flow diagram of the screening and selection process

The study selection process is summarized in Fig. 1. We retrieved 3156 records from the primary search strategy, of which 2903 were removed for non-clinical trials. Twenty-seven records were excluded for duplicate results. Two hundred and six records were further excluded due to retrospective studies, conference abstracts, case reports, reviews, not published in English, and mechanistic studies. Twenty studies were assessed for eligibility, and ten studies were excluded (combination therapy = 2, lack of study results = 4, identical published study = 2, others = 2). Ultimately, ten studies with 925 patients were enrolled in this meta-analysis.

### Study characteristics and quality assessment

The characteristics of the ten studies are shown in Table 1. Five studies used sotorasib [11–15], three studies used adagrasib [16, 18, 20], the other two studies used divarasib [19] and garsorasib [17], respectively. Seven studies reported NSCLC patients’ results [11, 12, 15–19], five studies reported CRC patients’ results [11, 14, 18–20], and four studies reported other (mostly pancreatic cancer and a variety of other tumors such as appendix, bile duct, endometrial, gastric and so on) patients’ results [11, 13, 18, 19]. The vast majority of studies included patients with a median No. of previous treatment lines ≥ 2. For NSCLC population, five studies were median No. of previous treatment lines = 2, the other one was 3. However, for CRC population, all four studies were median No. of previous treatment lines ≥ 3. And for other solid tumors, one study was median No. of previous treatment lines = 1, one was 2, and the other one was 3. Nine studies reported the results of median follow-up, and the shortest median follow-up time was 8.8 months (95% CI, 0.7–14.9).

**Table 1:** The characteristic of the studies included for the meta-analysis

Author Year	Study type	Drug	Patients	Median No. of previous lines (*n*)	No. (*n*)	ORR (%)	DCR (%)	PFS6 (%)	PFS12 (%)	OS6 (%)	OS 12 (%)	Any trAE (%)	≥ 3 trAE (%)
Hong 2020	phase 1	Sotorasib	NSCLC	-	59	32.2 (20.6–45.6)	88.1 (77.1–95.1)	50.8 (37.5–64.1)	23.7 (13.6–36.6)	-	-	-	-
			CRC	-	42	7.1 (1.5–19.5)	73.8 (58.0-86.1)	28.6 (15.7–44.6)	-	-	-	-	-
			Others	-	28	14.3 (4.0-32.7)	75.0 (55.1–89.3)	-	-	-	-	-	-
			total	3	129	-	-	-	-	-	-	56.6 (47.6–65.3)	12.4 (7.3–19.4)
Strickler 2023	phase 1–2	Sotorasib	Others	2	38	21.1 (9.6–37.3)	84.2 (68.7–94.0)	31.6 (17.5–48.7)	-	57.9 (40.8–73.7)	18.4(7.7–34.3)	42.1 (26.3–59.2)	15.8 (6.0-31.3)
Skoulidis 2021	phase 2	Sotorasib	NSCLC	2	126	36.5 (28.1–45.6)	79.4 (71.2–86.1)	52.4 (43.3–61.3)	27.0 (19.5–35.6)	75.4 (66.9–82.6)	51.6 (42.6–60.6)	69.8 (61.0-77.7)	20.6 (13.9–28.8)
Fakih 2022	phase 2	Sotorasib	CRC	3	62	9.7 (3.6–19.9)	82.3 (70.5–90.8)	22.6 (12.9–35.0)	11.3 (4.7–21.9)	83.9 (72.3–92.0)	41.9(29.5–55.2)	54.8 (41.7–67.5)	11.3 (4.7–21.9)
Langen 2023	phase 3	Sotorasib	NSCLC	2	171	28.1 (21.5–35.4)	82.5 (75.9–87.8)	46.8 (39.1–54.6)	24.6 (18.3–31.7)	71.9(64.6–78.5)	46.2(38.6–54.0)	69.6 (62.1–76.4)	32.7 (25.8–40.3)
Ou 2022	phase 1/1B	Adagrasib	NSCLC	3	16	50.0 (24.7–75.3)	100.0 (79.4–100.0)	62.5 (35.4–84.8)	50.0 (24.7–75.3)	75.0(47.6–92.7)	62.5(35.4–84.8)	-	-
			CRC	4	4	25.0 (0.6–80.6)	100.0 (39.8–100.0)	-	-	-	-	-	-
			Others	1	3	0.0 (0.0-70.8)	66.7 (9.4–99.2)	-	-	-	-	-	-
			total	-	23	-	-	-	-	-	-	92.0 (74.0–99.0)	36.0 (18.0-57.5)
Yaeger 2023	phase 1–2	Adagrasib	CRC	3	44	18.6 (8.3–33.4)	86.0 (72.1–94.7)	46.5 (31.2–62.3)	14.0 (5.3–27.9)	93.2(81.3–98.6)	68.2(52.4–81.4)	93.2 (81.3–98.6)	34.1 (20.5–49.9)
Jänne 2022	phase 2	Adagrasib	NSCLC	2	116	42.9 (33.5–52.6)	79.5 (70.8–86.5)	51.8 (42.1–61.3)	29.5 (21.2–38.8)	70.7(61.5–78.8)	50.9(41.4–60.3)	97.4 (92.6–99.5)	43.1 (33.9–52.6)
Sacher 2023	phase 1	Divarasib	NSCLC	2	60	60.3 (46.6–73.0)	89.7 (78.8–96.1)	72.4 (59.1–83.3)	55.2 (41.5–68.3)	-	-	-	-
			CRC	3	55	36.4 (23.8–50.4)	85.5 (73.3–93.5)	47.3(33.7–61.2)	16.4 (7.8–28.8)	-	-	-	-
			Others	3	22	36.4 (17.2–59.3)	86.4 (65.1–97.1)	-	-	-	-	-	-
			total	-	137	-	-	-	-	-	-	92.7 (87.0-96.4)	11.7 (6.8–18.3)
Li 2023	Phase 1	Garsorasib	NSCLC	2	79	40.5 (29.3–52.6)	91.9 (83.2–97.0)	67.6 (55.7–78.0)	32.4 (22.0-44.3)	-	-	94.9 (87.5–98.6)	38.0 (27.3–49.6)

The nine non-randomized controlled trials (RCT) studies without the control group scored 12–14 of 16, and the other RCT study scored 24 of 24 on MINORS (Supplement Table S1). The MINORS results suggested consistent high methodological quality for the included studies.

### Pooled analysis of ORR and DCR

**Fig. 2:**
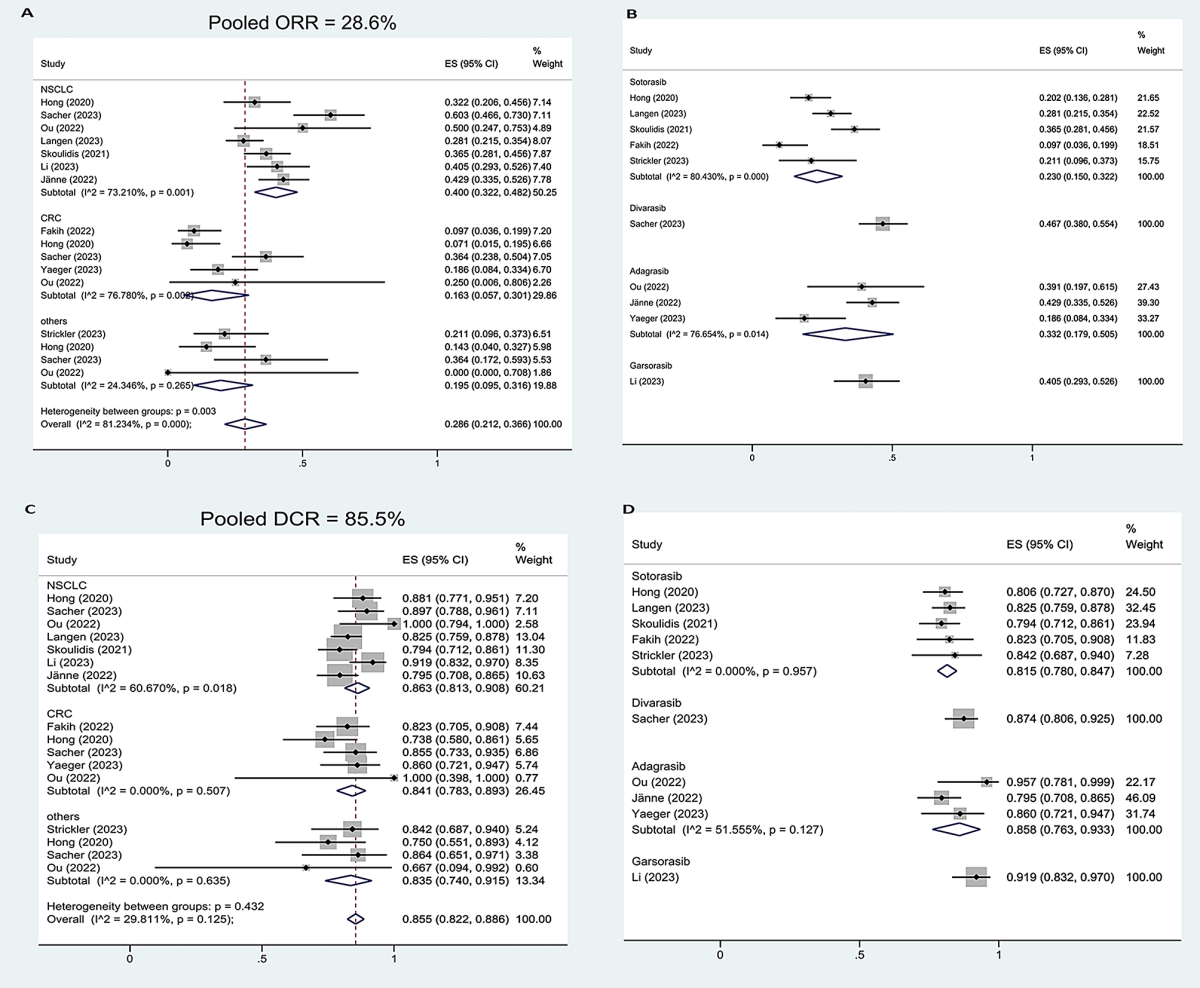
Pooled analysis of ORR and DCR: pooled ORR for total population and different cancers (**A**), pooled ORR for different drugs (**B**), pooled DCR for total population and different cancers (**C**), pooled DCR for different drugs (**D**)

The pooled results of ORR were 28.6% (95%CI, 21.2-36.6%, I^2^ = 81.23%) for total population, 40.0% (95%CI, 32.2-48.2%, I^2^ = 73.21%) for NSCLC patients, 16.3% (95%CI, 5.7-30.1%, I^2^ = 76.78%) for CRC patients, 19.5% (95%CI, 9.5-31.6%, I^2^ = 24.35%) for other cancers (Fig. 2A), 23.0% (95%CI, 15.0-32.2%, I^2^ = 80.43%) for sotorasib monotherapy, 33.2% (95%CI, 17.9-50.5%, I^2^ = 76.65%) for adagrasib monotherapy (Fig. 2B), 36.3% (95%CI, 26.9-46.2%, I^2^ = 78.15%) for median NO. of previous treatment lines ≤ 2, and 26.7% (95%CI, 13.6-41.9%, I^2^ = 75.14%) for median NO. of previous treatment lines > 2 (Supplement Figure S1A). NSCLC had significantly higher pooled ORR compared with CRC (OR, 3.36, 95%CI, 2.56–5.02, *p* < 0.0001) and other cancers (OR, 2.70, 95%CI, 1.58–4.60, *p* = 0.0002). Sotorasib had significantly lower pooled ORR compared with adagrasib (OR, 0.60, 95%CI, 0.42–0.87, *p* = 0.0073). The patients with median NO. of previous treatment lines ≤ 2 had significantly higher pooled ORR compared with > 2 lines (OR, 1.56, 95%CI, 1.09–2.22, *p* = 0.0137, Table 2).

**Table 2:** Comparison between subgroups for ORR, DCR, PFS rate, OS rate, and trAEs

	Subgroup	Pooled results (%) (95% CI)	Subgroup	Pooled results (%) (95% CI)	OR (95% CI)	*P*-value
ORR	NSCLC	40.0 (32.2–48.2)	CRC	16.3 (5.7–30.1)	3.36 (2.56–5.02)	< 0.0001
	NSCLC	40.0 (32.2–48.2)	Others	19.5 (9.5–31.6)	2.70 (1.58–4.60)	0.0002
	Sotorasib	23.0 (15.0-32.2)	Adagrasib	33.2 (17.9–50.5)	0.60 (0.42–0.87)	0.0073
	≤ 2 lines	36.3 (26.9–46.2)	> 2 lines	26.7 (13.6–41.9)	1.56 (1.09–2.22)	0.0137
DCR	NSCLC	86.3 (81.3–90.8)	CRC	84.1 (78.3–89.3)	1.21 (0.78–1.87)	0.397
	NSCLC	86.3 (81.3–90.8)	Others	83.5 (74.0-91.5)	1.25 (0.69–2.26)	0.465
	Sotorasib	81.5 (78.0-84.7)	Adagrasib	85.8 (76.3–93.3)	0.72 (0.45–1.16)	0.18
	≤ 2 lines	85.4 (80.9–89.4)	> 2 lines	88.5 (82.5–93.6)	0.75 (0.46–1.22)	0.25
PFS6	NSCLC	56.7 (49.3–64.0)	CRC	35.7 (23.4–49.0)	2.36 (1.70–3.28)	< 0.0001
	Sotorasib	39.4 (29.6–49.6)	Adagrasib	51.5 (43.8–59.1)	0.61 (0.43–0.87)	0.0057
	≤ 2 lines	54.1 (44.5–63.6)	> 2 lines	42.6 (26.6–59.3)	1.58 (1.13–2.23)	0.0078
PFS12	NSCLC	32.3 (24.9–40.2)	CRC	13.7 (8.6–19.6)	2.97 (1.93–4.56)	< 0.0001
	Sotorasib	22.0 (16.0-28.7)	Adagrasib	28.0 (13.2–45.5)	0.73 (0.49–1.08)	0.11
	≤ 2 lines	32.5 (24.1–41.6)	> 2 lines	18.8 (8.7–31.3)	2.09 (1.38–3.17)	0.0005
OS6	NSCLC	73.0 (68.6–77.2)	CRC	88.1 (81.1–93.8)	0.38 (0.20–0.69)	0.0015
	Sotorasib	73.6 (65.4–81.1)	Adagrasib	80.9 (62.0-94.7)	0.67 (0.43–1.03)	0.066
	≤ 2 lines	71.2 (66.0-76.2)	> 2 lines	86.4 (75.9–94.5)	0.40 (0.23–0.69)	0.0008
OS12	NSCLC	49.6 (44.8–54.5)	CRC	53.0 (43.3–62.5)	0.88 (0.58–1.35)	0.56
	Sotorasib	40.7 (29.5–52.4)	Adagrasib	58.9 (46.4–70.9)	0.48 (0.33–0.68)	0.0001
	≤ 2 lines	43.1 (32.3–54.3)	> 2 lines	56.7 (37.9–74.7)	0.58 (0.39–0.87)	0.0077
Any trAEs	Sotorasib	60.3 (51.2–69.0)	Adagrasib	95.9 (91.3–99.0)	0.07 (0.03–0.14)	< 0.0001
≥grade 3 trAEs	Sotorasib	18.5 (10.8–27.6)	Adagrasib	39.9 (32.8–47.2)	0.34 (0.24–0.49)	< 0.0001

The pooled results of DCR were 85.5% (95%CI, 82.2-88.6%, I^2^ = 29.81%) for total population, 86.3% (95%CI, 81.3-90.8%, I^2^ = 60.67%) for NSCLC patients, 84.1% (95%CI, 78.3-89.3%, I^2^ = 0.00%) for CRC patients, 83.5% (95%CI, 74.0-91.5%, I^2^ = 0.00%) for other cancers (Fig. 2C), 81.5% (95%CI, 78.0-84.7%, I^2^ = 0.00%) for sotorasib monotherapy, 85.8% (95%CI, 76.3-93.3%, I^2^ = 51.56%) for adagrasib monotherapy (Fig. 2D), 85.4% (95%CI, 80.9-89.4%, I^2^ = 37.64%) for median NO. of previous treatment lines ≤ 2, and 88.5% (95%CI, 82.5-93.6%, I^2^ = 14.52%) for median NO. of previous treatment lines > 2 (Supplement Figure S1B). The pooled DCR was not significantly different between NSCLC and CRC (OR, 1.21, 95%CI, 0.78–1.87, *p* = 0.397), NSCLC and others (OR, 1.25, 95%CI, 0.69–2.26, *p* = 0.465), sotorasib and adagrasib (OR, 0.72, 95%CI, 0.45–1.16, *p* = 0.18), median NO. of previous treatment lines ≤ 2 and > 2 (OR, 0.75, 95%CI, 0.46–1.22, *p* = 0.25, Table 2).

### Pooled analysis of time to response and DOR

There were 221 individual data of time to response and DoR were extracted. The median time to response was 1.39 months (95%CI, 1.37–1.41 months) for total population, 1.41 months (95%CI, 1.38–1.44 months) for sotorasib monotherapy, 1.36 months (95%CI, 1.33–1.39 months) for adagrasib monotherapy, and 1.38 months (95%CI, 1.33–1.43 months) for garsorasib monotherapy.

**Fig. 3:**
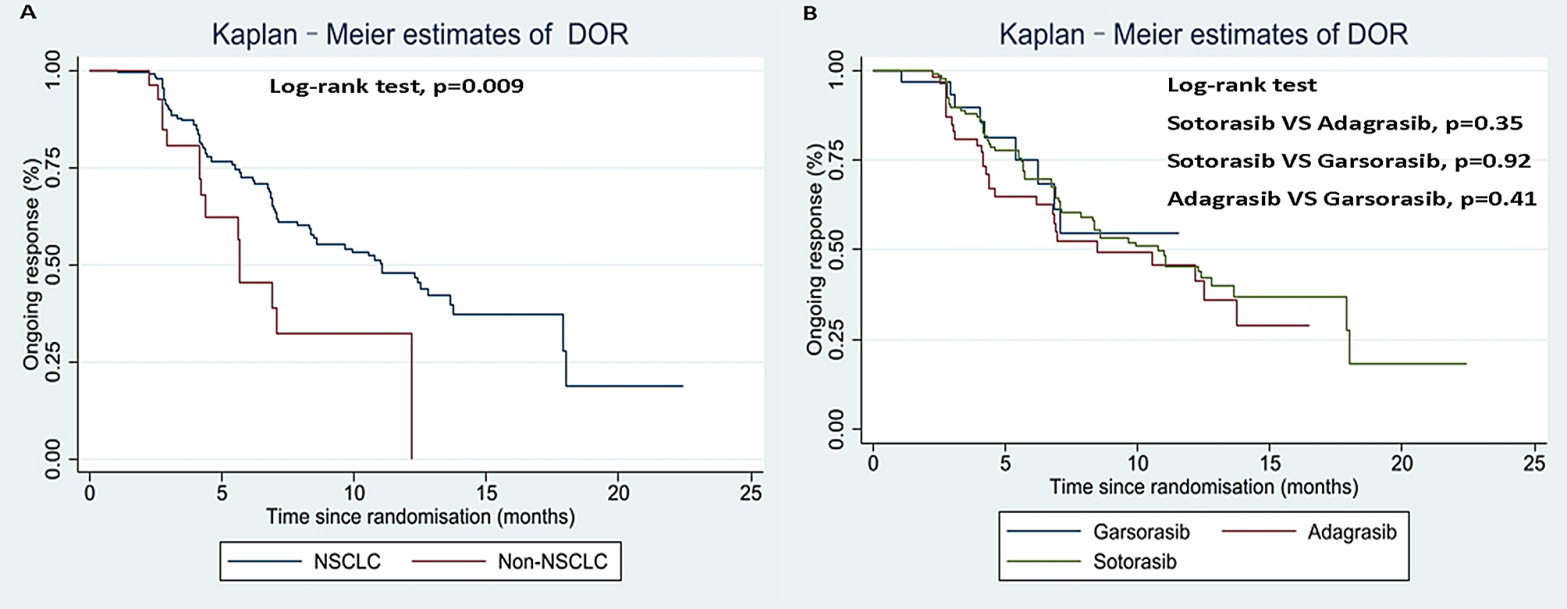
Kaplan-Meier estimates for extracted individual data of DoR by different tumors (**A**) and drugs (**B**)

The median DoR was 10.54 months (95%CI, 7.72–13.36 months) for total population, 11.06 months (95%CI, 8.02–14.10 months) for NSCLC patients, 5.68 months (95%CI, 4.13–7.23 months) for non-NSCLC patients (Fig. 3A), 10.77 months (95%CI, 7.42–14.12 months) for sotorasib, 8.49 months (95%CI, 3.63–13.35 months) for adagrasib, not evaluable for garsorasib (Fig. 3B). NSCLC patients had significantly better DoR than non-NSCLC patients (Log-rank, *p* = 0.009). However, DoR was not significantly different among the three drugs (Log-rank, sotorasib VS adagrasib, *p* = 0.35; sotorasib VS garsorasib, *p* = 0.92; adagrasib VS garsorasib, *p* = 0.41).

### Pooled analysis of PFS

**Fig. 4:**
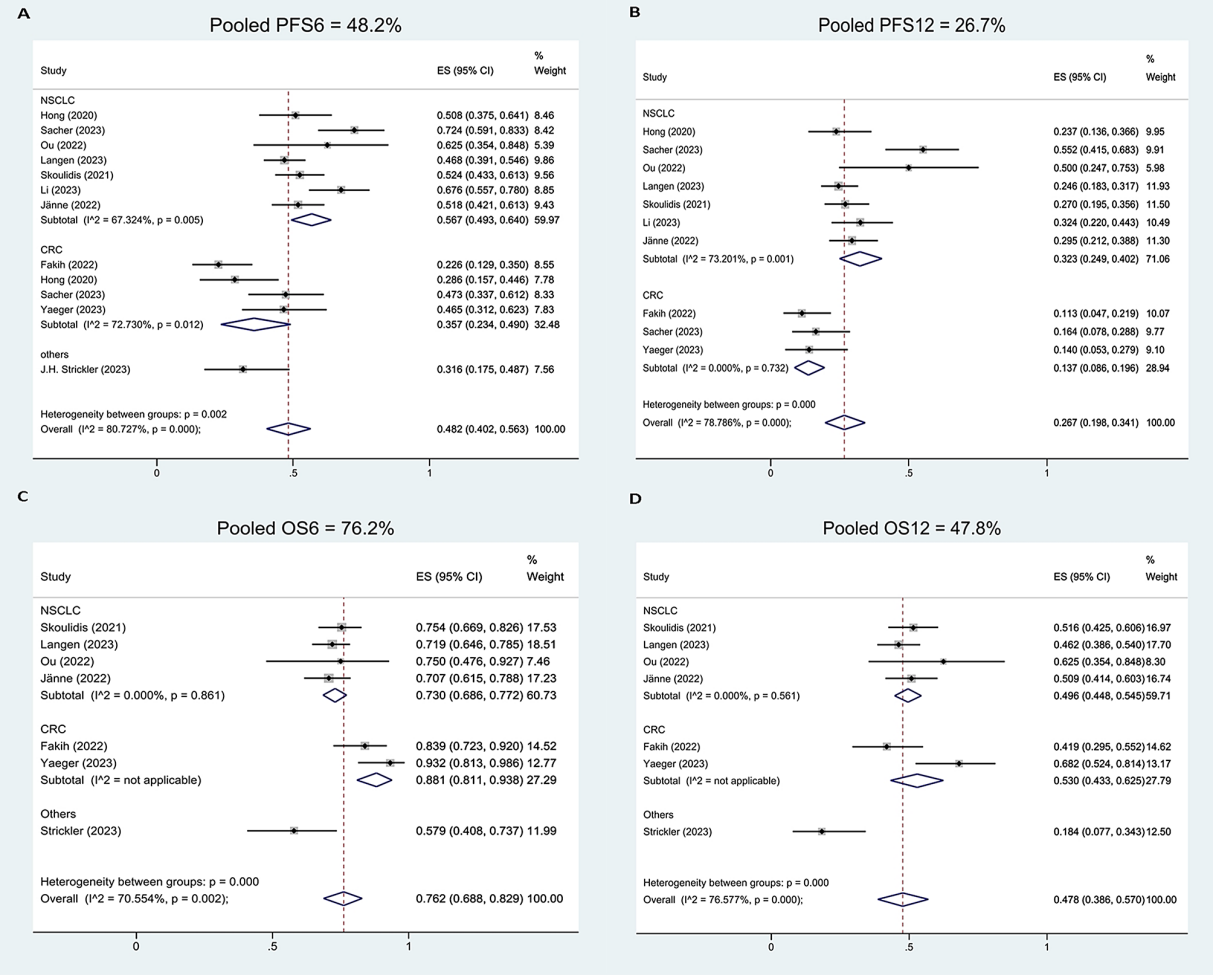
Pooled analysis of PFS rate at 6 months (**A**), PFS rate at 12 months (**B**), OS rate at 6 months (**C**), and OS rate at 12 months (**D**)

The pooled PFS rates at 6 months (PFS6) were 48.2% (95%CI, 40.2-56.3%, I^2^ = 80.73%) for total population, 56.7% (95%CI, 49.3-64.0%, I^2^ = 67.32%) for NSCLC patients, 35.7% (95%CI, 23.4-49.0%, I^2^ = 72.73%) for CRC patients (Fig. 4A), 39.4% (95%CI, 29.6-49.6%, I^2^ = 79.22%) for sotorasib monotherapy, 51.5% (95%CI, 43.8-59.1%, I^2^ = 0.00%) for adagrasib monotherapy (Supplement Figure S2A), 54.1% (95%CI, 44.5-63.6%, I^2^ = 80.30%) for median NO. of previous treatment lines ≤ 2, and 42.6% (95%CI, 26.6-59.3%, I^2^ = 78.02%) for median NO. of previous treatment lines > 2 (Supplement Figure S2B).The pooled PFS rates at 12 months (PFS12) were 26.7% (95%CI, 19.8-34.1%, I^2^ = 78.79%) for total population, 32.3% (95%CI, 24.9-40.2%, I^2^ = 73.20%) for NSCLC patients, 13.7% (95%CI, 8.6-19.6%, I^2^ = 0.00%) for CRC patients (Fig. 4B), 22.0% (95%CI, 16.0-28.7%, I^2^ = 56.90%) for sotorasib monotherapy, 28.0% (95%CI, 13.2-45.5%, I^2^ = 75.54%) for adagrasib monotherapy (Supplement Figure S2C), 32.5% (95%CI, 24.1-41.6%, I^2^ = 78.43%) for median NO. of previous treatment lines ≤ 2, and 18.8% (95%CI, 8.7-31.3%, I^2^ = 70.20%) for median NO. of previous treatment lines > 2 (Supplement Figure S2D).

NSCLC had significantly higher pooled PFS6 (OR, 2.36, 95%CI, 1.70–3.28, *p* < 0.0001) and PFS12 (OR, 2.97, 95%CI, 1.93–4.56, *p* < 0.0001) compared with CRC. Sotorasib had significantly lower pooled PFS6 compared with adagrasib (OR, 0.61, 95%CI, 0.43–0.87, *p* = 0.0057); however, the pooled PFS12 was not significantly different (OR, 0.73, 95%CI, 0.49–1.08, *p* = 0.11). The patients with median NO. of previous treatment lines ≤ 2 had significantly higher pooled PFS6 (OR, 1.58, 95%CI, 1.13–2.23, *p* = 0.0078) and PFS12 (OR, 2.09, 95%CI, 1.38–3.17, *p* = 0.0005) compared with > 2 lines (Table 2).

### Pooled analysis of OS

The pooled OS rates at 6 months (OS6) were 76.2% (95%CI, 68.8-82.9%, I^2^ = 70.55%) for total population, 73.0% (95%CI, 68.6-77.2%, I^2^ = 0.00%) for NSCLC patients, 88.1% (95%CI, 81.1-93.8%, I^2^ = not applicable) for CRC patients (Fig. 4C), 73.6% (95%CI, 65.4-81.1%, I^2^ = 64.17%) for sotorasib monotherapy, 80.9% (95%CI, 62.0-94.7%, I^2^ = 82.01%) for adagrasib monotherapy (Supplement Figure S3A), 71.2% (95%CI, 66.0-76.2%, I^2^ = 28.19%) for median NO. of previous treatment lines ≤ 2, and 86.4% (95%CI, 75.9-94.5%, I^2^ = 47.16%) for median NO. of previous treatment lines > 2 (Supplement Figure S3B). The pooled OS rates at 12 months (OS12) were 47.8% (95%CI, 38.6-57.0%, I^2^ = 76.58%) for total population, 49.6% (95%CI, 44.8-54.5%, I^2^ = 0.00%) for NSCLC patients, 53.0% (95%CI, 43.3-62.5%, I^2^ = not applicable) for CRC patients (Fig. 4D), 40.7% (95%CI, 29.5-52.4%, I^2^ = 79.55%) for sotorasib monotherapy, 58.9% (95%CI, 46.4-70.9%, I^2^ = 51.23%) for adagrasib monotherapy (Supplement Figure S3C), 43.1% (95%CI, 32.3-54.3%, I^2^ = 80.94%) for median NO. of previous treatment lines ≤ 2, and 56.7% (95%CI, 37.9-74.7%, I^2^ = 73.59%) for median NO. of previous treatment lines > 2 (Supplement Figure S3D).

NSCLC had significantly lower pooled OS6 (OR, 0.38, 95%CI, 0.20–0.69, *p* = 0.0015) compared with CRC; however, the pooled OS12 was not significantly different (OR, 0.88, 95%CI, 0.58–1.35, *p* = 0.56). Sotorasib had significantly lower pooled OS12 compared with adagrasib (OR, 0.48, 95%CI, 0.33–0.68, *p* = 0.0001); however, the pooled OS6 was not significantly different (OR, 0.67, 95%CI, 0.43–1.03, *p* = 0.066). The patients with median NO. of previous treatment lines ≤ 2 had significantly lower pooled OS6 (OR, 0.40, 95%CI, 0.23–0.69, *p* = 0.0008) and OS12 (OR, 0.58, 95%CI, 0.39–0.87, *p* = 0.0077) compared with > 2 lines (Table 2).

### Pooled analysis of trAEs

**Fig. 5:**
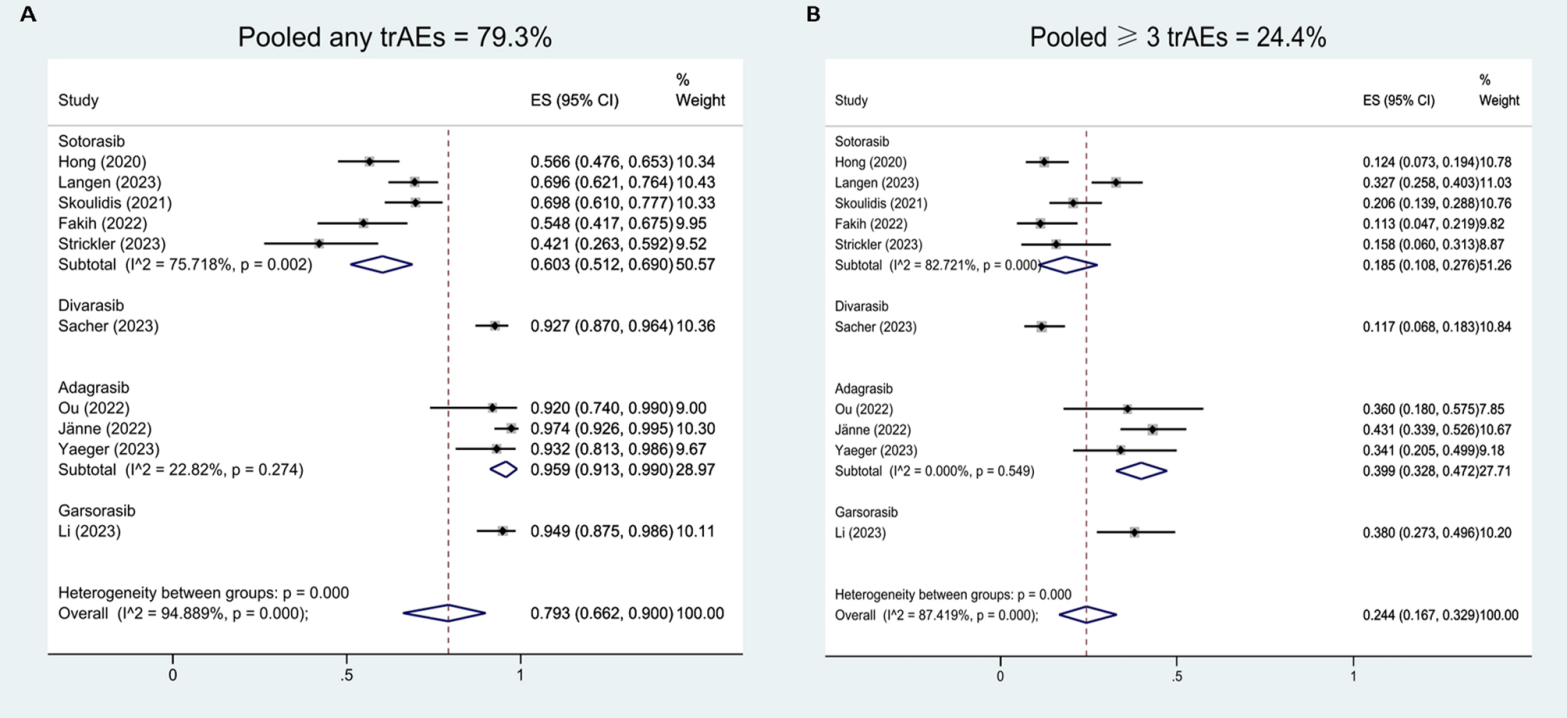
Pooled incidences of any trAEs (**A**) and grade three or more trAEs (**B**)

The pooled incidences of any grade trAEs were 79.3% (95%CI, 66.2-90.0%, I^2^ = 94.89%) for total population, 60.3% (95%CI, 51.2-69.0%, I^2^ = 75.72%) for sotorasib monotherapy, 95.9% (95%CI, 91.3-99.0%, I^2^ = 22.82%) for adagrasib monotherapy (Fig. 5A). The pooled incidences of grade three or more trAEs were 24.4% (95%CI, 16.7-32.9%, I^2^ = 87.42%) for total population, 18.5% (95%CI, 10.8-27.6%, I^2^ = 82.72%) for sotorasib monotherapy, 39.9% (95%CI, 32.8-47.2%, I^2^ = 0.00%) for adagrasib monotherapy (Fig. 5B). Sotorasib had significantly lower pooled incidences of any trAES (OR, 0.07, 95%CI, 0.03–0.14, *p* < 0.0001) and grade three or more trAEs (OR, 0.34, 95%CI, 0.24–0.49, *p* < 0.0001) compared with adagrasib (Table 2).

There were 20 specific trAEs which were reported in at least three studies (Supplement Table S2). Diarrhoea, nausea, vomiting, fatigue, ECG QT prolonged, aspartate aminotransferase (AST) increased, alanine aminotransferase (ALT) increased, amylase increase, blood creatinine increase/acute kidney injury, decreased appetite, and anaemia were the very common (≥ 10%) any grade trAEs. Alkaline phosphatase increased, lipase increased, dysgeusia, edema peripheral, hyponatremia, abdominal pain, pneumonitis, lymphocyte count decrease, and neutrophil count decrease were the common (< 10%, ≥ 1%) any grade trAEs.

ALT increased, AST increased, diarrhoea, ECG QT prolonged, lipase increased, anaemia, fatigue, alkaline phosphatase increased, amylase increased, and hyponatremia were the common grade three or more trAEs. Blood creatinine increase/acute kidney injury, pneumonitis, lymphocyte count decrease, decreased appetite, nausea, neutrophil count decrease, abdominal pain, and vomiting were the uncommon (< 1%, ≥ 0.1%) grade three or more trAEs (Supplement Table S3). The other reported grade three or more specific trAEs were rare (< 0.1%, ≥ 0.01%) or very rare (< 0.01%).

Subgroup analyses for the incidences of the 20 specific trAEs by different drugs are also shown in Table S2 and Table S3. Most of the any grade specific trAEs were more common for adagrasib compared with sotorasib. However, for the grade three or more specific trAEs, some were more common for adagrasib while some others were not.

### Publication bias analysis

The publication bias was analyzed by Egger’s test. The p-values of ORR, DCR, PFS6, PFS12, OS6, OS12, incidence of any trAEs, and incidence of grade three or more trAEs were 0.454, 0.402, 0.740, 0.671, 0.676, 0.996, 0.974, and 0.846, respectively (Supplement Figure S4). These results did not indicate any publication bias.

## Discussion

KRAS mutations are common in a variety of cancers, and they are considered to have some impact on the prognoses of cancer patients [23–25]. Specific KRAS^G12C^ mutation inhibitors such as sotorasib, adagrasib, divarasib, and garsorasib have been evaluated in recent years and have shown encouraging results. A recent meta-analysis reported the pooled results for KRAS^G12C^ inhibitors in solid tumors. However, that study included combination therapy as well as monotherapy and only ORR, DCR, and ≥ grade three AEs were reported for KRAS^G12C^ inhibitors monotherapy [26]. Our study suggested that KRAS^G12C^ inhibitors monotherapy had good ORR, DCR, PFS rate, OS rate, tolerable trAEs, and early response with long duration in advanced solid tumors.

For total population, the pooled analysis of ORR was 28.6% (95%CI, 21.2-36.6%), DCR was 85.5% (95%CI, 82.2-88.6%), PFS rate at 6 months (PFS6) was 49.6% (95%CI, 41.4-57.9%), PFS rate at 12 months (PFS12) was 26.7% (95%CI, 19.8-34.1%), OS rates at 6 months (OS6) was 76.2% (95%CI, 68.8-82.9%), OS rates at 12 months (OS12) was 47.8% (95%CI, 38.6-57.0%). The pooled analysis of any grade trAEs was 79.3% (95%CI, 66.2-90.0%), and grade three or more trAEs was 24.4% (95%CI, 16.7-32.9%). The median time to response and DoR results from individual data were 1.39 months (95%CI, 1.37–1.41 months) and 10.54 months (95%CI, 7.72–13.36 months). It should be noted that most of the studies included in the meta-analysis enrolled previously heavily treated patients who were prone to resist systemic therapy and lack of treatment options. Therefore, the pooled results of our study indicated that KRAS^G12C^ inhibitors had good ORR, DCR, PFS rate, OS rate, tolerable trAEs, and early response with long duration. These results supported the use of KRAS^G12C^ inhibitors in advanced solid tumors.

For subgroup analysis by cancer types, our study suggested that NSCLC had significantly better pooled ORR, DoR, and PFS rate compared with CRC and/or others. However, the pooled OS6 was significantly lower compared with CRC. The pooled results for NSCLC were supported by a recent phase 3 RCT study that advanced NSCLC with KRAS^G12C^ mutation who progressed after previous platinum-based chemotherapy and immune checkpoint inhibitors were randomly assigned (1:1) to oral sotorasib or intravenous docetaxel [15]. In this study, the ORR, DCR, and median DoR were 28.1%, 82.5%, and 8.6 months for sotorasib group, and 13.2%, 60.3%, and 6.8 months for docetaxel group. The primary endpoint PFS was statistically significantly increased by sotorasib with fewer grade three or more trAEs compared with docetaxel. However, the median PFS improvement was modest clinically meaningful (from 4.5 months to 5.6 months), and the OS was not significantly different (HR, 1.01, 95%CI, 0.77–1.33). These results led to questionable efficacy of sotorasib for NSCLC with KRAS^G12C^ mutation [27]. Despite negative OS results in this phase III trial, sotorasib still had higher ORR and DCR, longer DoR, and fewer trAEs compared with docetaxel. It should also be noted that this study was not powered for OS and there were 33.9% patients in the chemotherapy group crossed over to sotorasib group. Further studies are needed to select suitable patients for KRAS^G12C^ inhibitors. Another ongoing phase 3 RCT study (NCT04685135) evaluating adagrasib versus docetaxel in advanced NSCLC with KRAS^G12C^ mutation may provide more evidences for whether KRAS^G12C^ inhibitors are superior to standard care therapy (SOC). Despite the encouraging results for KRAS^G12C^ inhibitors monotherapy in NSCLC, the results in CRC and other cancers were less satisfactory. KRAS^G12C^ inhibitors combined therapy was investigated in another phase 3 RCT study that chemorefractory metastatic CRC patients with KRAS^G12C^ mutation were randomly assigned in a 1:1:1 ratio to receive sotorasib (960 mg once daily) plus panitumumab, sotorasib (240 mg once daily) plus panitumumab, and the investigator’s choice of SOC [28]. The ORR, DCR, and median DoR were 26.4%, 71.7%, and 4.4 months for sotorasib (960 mg) combination group, 5.7%, 67.9%, and not estimated for sotorasib (240 mg) combination group, 0%, 46.3%, and not estimated for SOC group. The primary endpoint PFS was also significantly increased by sotorasib (960 mg) combination (HR, 0.49, 95%CI, 0.30 to 0.80; *P* = 0.006) and sotorasib (240 mg) combination (HR, 0.58,95%CI, 0.36 to 0.93; *P* = 0.03) compared with SOC group. KRAS^G12C^ inhibitor plus an EGFR inhibitor had better efficacy than SOC in CRC; however, this combination therapy seemed to have similar or even worse ORR, DCR, and DoR compared with our pooled results. EGFR inhibitor was considered to be ineffective for RAS mutated CRC [29, 30]. Whether this combination therapy is superior compared with KRAS^G12C^ inhibitor monotherapy is still unclear. Further studies are required to investigate the reasonable combination therapy strategy for KRAS^G12C^ mutated solid tumors beyond NSCLC. Regarding other tumors, pancreatic cancer is another common solid tumor with KRAS^G12C^ mutation. A phase 1–2 study assessed sotorasib in pancreatic cancer who had received at least one previous systemic therapy and showed anticancer activity. The other three phase 1 studies assessed sotorasib, adagrasib, and divarasib in solid tumors other than NSCLC and CRC. However, due to fewer patients included and early phase of the trials, the PFS and OS results could not be pooled. The pooled ORR seemed to be similar with CRC. KRAS^G12C^ inhibitor might be an option for these patients.

For subgroup analysis by drugs, our study suggested that sotorasib had significantly lower pooled ORR, PFS6, and OS12 compared with adagrasib; however, the DCR, DoR, PFS12, and OS6 were not significantly different. Sotorasib also had significantly lower pooled incidences of any trAEs and grade three or more trAEs. And most of the any grade specific trAEs were more common for adagrasib compared with sotorasib. The mean half-life was 5.5 h for sotorasib and 23 h for adagrasib [11, 18], and sotorasib was applied once daily while adagrasib was applied twice daily. Therefore, we speculated that adagrasib might have more accumulated toxicity than sotorasib due to its longer half-life and more frequency of administration. The accumulated dose of adagrasib might also be one of the reasons for higher pooled ORR compared with sotorasib because high dose of KRAS^G12C^ inhibitor seemed to have better ORR than low dose in a CRC phase 3 RCT study [28]. However, it should be noted that adagrasib included a relatively higher proportion of NSCLC (72.1% VS 67.7%) whose ORR is better than non-NSCLC and only phase 1–2 study with fewer patients (183 VS 526) which might be more heterogeneous. Therefore, the efficacy conclusions should be made carefully between the two drugs. Divarasib and garsorasib were also assessed in phase 1 study. Both drugs seemed to have similar ORR and DCR compared with adagrasib. Regarding safety profiles, divarasib seemed to have a higher incidence of any trAEs but similar grade three or more trAEs compared with sotorasib. And garsorasib seemed to have similar incidences of any trAEs and grade three or more trAEs compared with adagrasib. However, no conclusion could be indicated because there was only one phase 1 study for each drug. Further studies are needed to evaluate the efficacy and toxicity results among different KRAS^G12C^ inhibitors.

For subgroup analysis by previous treatment lines, the patients with median NO. of previous lines ≤ 2 had significantly higher pooled ORR, PFS6, and PFS12 compared with > 2 lines; however, the pooled OS6 and OS12 were significantly lower for ≤ 2 lines. We should note that most patients with median NO. of previous lines ≤ 2 were NSCLC and most patients with > 2 lines were CRC. Therefore, it is hard to say whether these differences were caused by different tumors or previous treatment lines.

Most of our pooled results were heterogeneous even for subgroup analysis by different cancers and drugs. The heterogeneity might be mainly due to the non-RCT studies with different baseline characteristics of the patients such as race, number of lines of previous anticancer therapy, previous drugs used, and co-mutation status. KEAP1 co-mutations were prone to have poorer outcomes for patients treated with KRAS^G12C^ inhibitors and were considered to be associated with resistance [1, 26]. RTK-RAS pathway and switch-II binding pocket mutations might be the mechanisms of acquired resistance to KRAS^G12C^ inhibitors [31]. KRAS^G12C^ inhibitors combination therapy and pan-RAS inhibitors may help to improve efficacy for patients with KRAS^G12C^ mutation.

We conducted a comprehensive analysis of the efficacy and toxicity characteristics of KRAS^G12C^ inhibitors monotherapy in advanced solid tumors. Our pooled results contained ORR, DCR, DoR, PFS rate, OS rate, and incidences of any trAEs, grade three or more trAEs, and specific trAEs. These results may help us to understand more about the characteristics of this treatment. However, our study had limitations. Firstly, most of our pooled results were heterogeneous, and the sources of heterogeneity were still unclear. Additionally, the comparison among different subgroups was not head-to-head, and further studies are required to confirm these results. Furthermore, most of the included trials were phase 1 or 2 non-RCT studies which might cause biases and heterogeneities.

## Conclusions

KRAS^G12C^ inhibitors had good ORR, DCR, PFS rate, OS rate, tolerable trAEs, and early response with long duration in advanced solid tumors; however, most of the pooled results were heterogeneous. NSCLC had significantly better pooled ORR, DoR, and PFS rate compared with CRC and/or others. However, the pooled OS6 was significantly lower compared with CRC. Sotorasib had significantly lower pooled ORR, PFS6, and OS12 compared with adagrasib; however, the DCR, DoR, PFS12, and OS6 were not significantly different. Sotorasib also had significantly lower pooled incidences of any trAEs and grade three or more trAEs compared with adagrasib. The patients with median NO. of previous lines ≤ 2 had significantly higher pooled ORR, PFS6, and PFS12 compared with > 2 lines; however, the pooled OS6 and OS12 were significantly lower for ≤ 2 lines.

### Electronic supplementary material

Below is the link to the electronic supplementary material.

Supplementary Material 1

## Data Availability

The data underlying this article are available in the article and in its supplementary material.

## Abbreviations

KRAS: Kirsten rat sarcoma.
MAPK: Mitogen activated protein kinase
PI3K: Phosphatidylinositol 3-kinase
NSCLC: Non-small-cell lung cancer
CRC: Colorectal cancers
MeSH: Medical Subject Headings
PRISMA: Preferred Reporting Items for Systematic Reviews and Meta-analyses
ORR: Objective response rate
DCR: Disease control rate
DoR: Duration of response
PFS: Progression-free survival
OS: Overall survival
trAEs: Treatment related adverse events
MINORS: Methodological Index for Nonrandomized Studies
OR: Odds ratio
RCT: Randomized controlled trial
PFS6: PFS rates at 6 months
PFS12: PFS rates at 12 months
PFS6: OS rates at 6 months
PFS12: OS rates at 12 months
AST: Aspartate aminotransferase
ALT: Alanine aminotransferase
