# Supplementary material for: Efficacy and toxicity of KRASG12C inhibitors in advanced solid tumors: a meta-analysis
Source: World J Surg Oncol. 2024 Jul 16;22:182. doi: 10.1186/s12957-024-03449-8 (PMC11251097; doi:10.1186/s12957-024-03449-8)
Supplement: Supplementary file 1 — Supplementary Material 1 [file 12957_2024_3449_MOESM1_ESM.docx]

Table S1. MINORS assessment for the studies.

|  | Hong | Strickler | Skoulidis | Fakih | Langen | Ou | Yaeger | Jänne | Sacher | Li |
| --- | --- | --- | --- | --- | --- | --- | --- | --- | --- | --- |
| A clearly stated aim | 2 | 2 | 2 | 2 | 2 | 2 | 2 | 2 | 2 | 2 |
| Inclusion of consecutive patients | 2 | 2 | 2 | 2 | 2 | 2 | 2 | 2 | 2 | 2 |
| Prospective collection of data | 2 | 2 | 2 | 2 | 2 | 2 | 2 | 2 | 2 | 2 |
| Endpoints appropriate to the aim of the study | 2 | 2 | 2 | 2 | 2 | 2 | 2 | 2 | 2 | 2 |
| Unbiased assessment of the study endpoint | 2 | 2 | 2 | 2 | 2 | 2 | 2 | 2 | 2 | 2 |
| Follow-up period appropriate to the aim of the study | 2 | 2 | 2 | 2 | 2 | 2 | 2 | 2 | 0 | 2 |
| Loss to follow up less than 5% | 2 | 2 | 2 | 2 | 2 | 2 | 2 | 2 | 2 | 2 |
| Prospective calculation of the study size | 0 | 0 | 0 | 0 | 2 | 0 | 0 | 0 | 0 | 0 |
|  |  |  |  |  |  |  |  |  |  |  |
| *Additional criteria in the case of comparative studies* | | | | | | | | | | |
| An adequate control group |  |  |  |  | 2 |  |  |  |  |  |
| Contemporary groups |  |  |  |  | 2 |  |  |  |  |  |
| Baseline equivalence of groups |  |  |  |  | 2 |  |  |  |  |  |
| Adequate statistical analyses |  |  |  |  | 2 |  |  |  |  |  |
| Total scores | 14 | 14 | 14 | 14 | 24 | 14 | 14 | 14 | 12 | 14 |

Table S2. Specific any trAEs which were reported in at least three studies.

| trAEs | Sotorasib  (%, 95%CI) | No. of studies | Adagrasib  (%, 95%CI) | No. of studies | Overall  (%, 95%CI) | No. of studies |
| --- | --- | --- | --- | --- | --- | --- |
| Diarrhoea | 24.05  (16.93-31.96) | 5 | 63.79  (56.11-71.15) | 2 | 35.78  (23.48-49.08) | 9 |
| Nausea | 11.36  (6.24-17.67) | 5 | 63.18  (54.56-71.41) | 3 | 31.77  (15.21-51.05) | 10 |
| Vomiting | 5.55  (3.51-7.98) | 3 | 47.01  (39.73-54.34) | 3 | 25.92  (10.34-45.37) | 8 |
| Fatigue | 7.40  (4.27-11.24) | 4 | 41.55  (34.42-48.86) | 3 | 19.63  (9.59-32.02) | 8 |
| ECG QT prolonged | no reported | 0 | 16.97  (11.73-22.90) | 3 | 16.97  (11.73-22.90) | 3 |
| Aspartate aminotransferase increased | 9.08  (5.01-14.11) | 4 | 20.82  (11.78-31.50) | 3 | 15.39  (8.69-23.48) | 9 |
| Alanine aminotransferase increased | 9.54  (6.39-13.19) | 5 | 20.02  (10.46-31.56) | 3 | 14.74  (8.42-22.36) | 9 |
| Amylase increase | no reported | 0 | 15.13  (9.47-21.73) | 2 | 14.67  (10.15-19.81) | 3 |
| Blood creatinine increase/Acute kidney injury | 1.61  (0.04-8.66) | 1 | 26.00  (18.92-33.72) | 2 | 14.31  (3.63-29.82) | 4 |
| Decreased appetite | 7.75  (4.94-11.10) | 2 | 22.37  (16.15-29.25) | 2 | 13.17  (7.99-19.36) | 6 |
| Anaemia | 4.60  (2.00-8.06) | 3 | 18.08  (12.69-24.13) | 3 | 12.94  (5.94-21.94) | 7 |
| Alkaline phosphatase increased | 5.85  (3.80-8.26) | 4 | 10.86  (6.01-16.78) | 2 | 9.06  (5.06-13.99) | 7 |
| Lipase increased | 0.79  (0.02-4.34) | 1 | 13.81  (8.38-20.22) | 2 | 8.32  (1.50-19.12) | 4 |
| Dysgeusia | 2.34  (0.64-5.88) | 1 | 10.86  (6.01-16.78) | 2 | 7.51  (1.19-17.73) | 3 |
| Edema peripheral | 0.00  (0.00-2.13) | 1 | 11.51  (7.10-16.72) | 3 | 7.07  (0.06-21.27) | 4 |
| Hyponatremia | 0.78  (0.02-4.24) | 1 | 10.34  (5.46-17.37) | 1 | 6.59  (0.59-17.45) | 3 |
| Abdominal pain | 4.83  (2.67-7.51) | 3 | 12.00  (2.55-31.22) | 1 | 5.01  (2.82-7.69) | 4 |
| Pneumonitis | 0.37  (0.00-1.58) | 2 | 5.73  (2.18-10.50) | 2 | 2.46  (0.00-7.98) | 4 |
| Lymphocyte count decrease | 1.94  (0.49-4.14) | 2 | 1.72  (0.21-6.09) | 1 | 1.87  (0.63-3.62) | 3 |
| Neutrophil count decrease | 0.90  (0.00-3.36) | 2 | 3.45  (0.95-8.59) | 1 | 1.82  (0.33-4.12) | 3 |

Table S3. Specific grade three or more trAEs which were reported in at least three studies.

| trAEs | Sotorasib  (%, 95%CI) | No. of studies | Adagrasib  (%, 95%CI) | No. of studies | Overall  (%, 95%CI) | No. of studies |
| --- | --- | --- | --- | --- | --- | --- |
| Alanine aminotransferase increased | 5.40  (3.53-7.60) | 5 | 4.00  (1.36-7.64) | 3 | 5.43  (3.85-7.22) | 9 |
| Aspartate aminotransferase increased | 4.26  (2.35-6.60) | 4 | 3.43  (0.99-6.89) | 3 | 4.13  (2.75-5.74) | 9 |
| Diarrhoea | 5.52  (2.64-9.26) | 5 | 1.82  (0.12-4.80) | 2 | 4.03  (2.01-6.61) | 9 |
| ECG QT prolonged | no reported | 0 | 4.00  (1.36-7.64) | 3 | 4.00  (1.36-7.64) | 3 |
| Lipase increased | 0.79  (0.02-4.34) | 1 | 5.25  (1.86-9.89) | 2 | 3.01  (0.67-6.52) | 4 |
| Anaemia | 1.03  (0.00-3.21) | 3 | 5.52  (2.41-9.57) | 3 | 2.91  (0.93-5.69) | 7 |
| Fatigue | 0.73  (0.00-2.92) | 4 | 4.89  (1.90-8.89) | 3 | 1.87  (0.31-4.30) | 8 |
| Alkaline phosphatase increased | 0.87  (0.00-2.79) | 4 | 2.79  (0.40-6.60) | 2 | 1.45  (0.29-3.20) | 7 |
| Amylase increase | no reported | 0 | 0.83  (0.00-3.61) | 2 | 1.36  (0.06-3.71) | 3 |
| Hyponatremia | 0.78  (0.02-4.24) | 1 | 4.31  (1.41-9.77) | 1 | 1.24  (0.00-4.43) | 3 |
| Blood creatinine increase/Acute kidney injury | 1.61  (0.04-8.66) | 1 | 0.31  (0.00-2.60) | 2 | 0.79  (0.00-2.53) | 4 |
| Pneumonitis | 0.37  (0.00-1.58) | 2 | 1.61  (0.00-4.87) | 2 | 0.76  (0.00-2.93) | 4 |
| Lymphocyte count decrease | 0.25  (0.00-1.50) | 2 | 1.72  (0.21-6.09) | 1 | 0.60  (0.00-2.02) | 3 |
| Decreased appetite | 0.70  (0.00-2.12) | 2 | 1.96  (0.17-5.01) | 2 | 0.47  (0.00-1.75) | 6 |
| Nausea | 0.10  (0.00-0.80) | 5 | 1.28  (0.00-5.18) | 3 | 0.41  (0.00-1.22) | 10 |
| Neutrophil count decrease | 0.00  (0.00-0.94) | 2 | 1.72  (0.21-6.09) | 1 | 0.28  (0.00-1.87) | 3 |
| Abdominal pain | 0.51  (0.00-2.47) | 3 | 0.00  (0.00-13.72) | 1 | 0.26  (0.00-1.55) | 4 |
| Vomiting | 0.00  (0.00-0.48) | 3 | 0.19  (0.00-1.96) | 3 | 0.11  (0.00-0.66) | 8 |
| Dysgeusia | 0.00  (0.00-2.13) | 1 | 0.00  (0.00-0.91) | 2 | 0.00  (0.00-0.33) | 3 |
| Edema peripheral | 0.00  (0.00-2.13) | 1 | 0.00  (0.00-0.78) | 3 | 0.00  (0.00-0.30) | 4 |


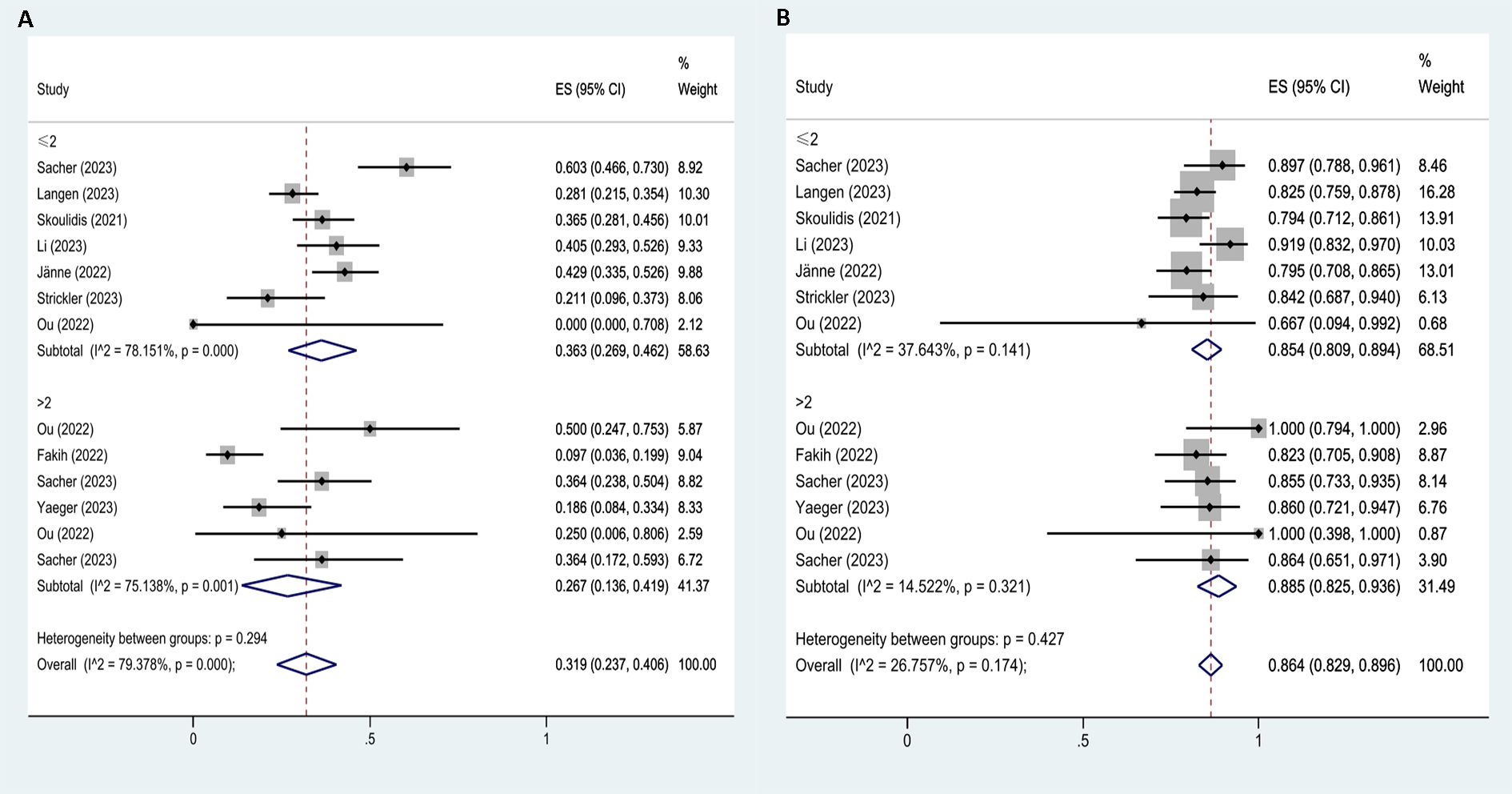


Figure S1. Subgroup analysis for median NO. of previous treatment lines ≤ 2 and > 2: ORR (A), DCR (B).


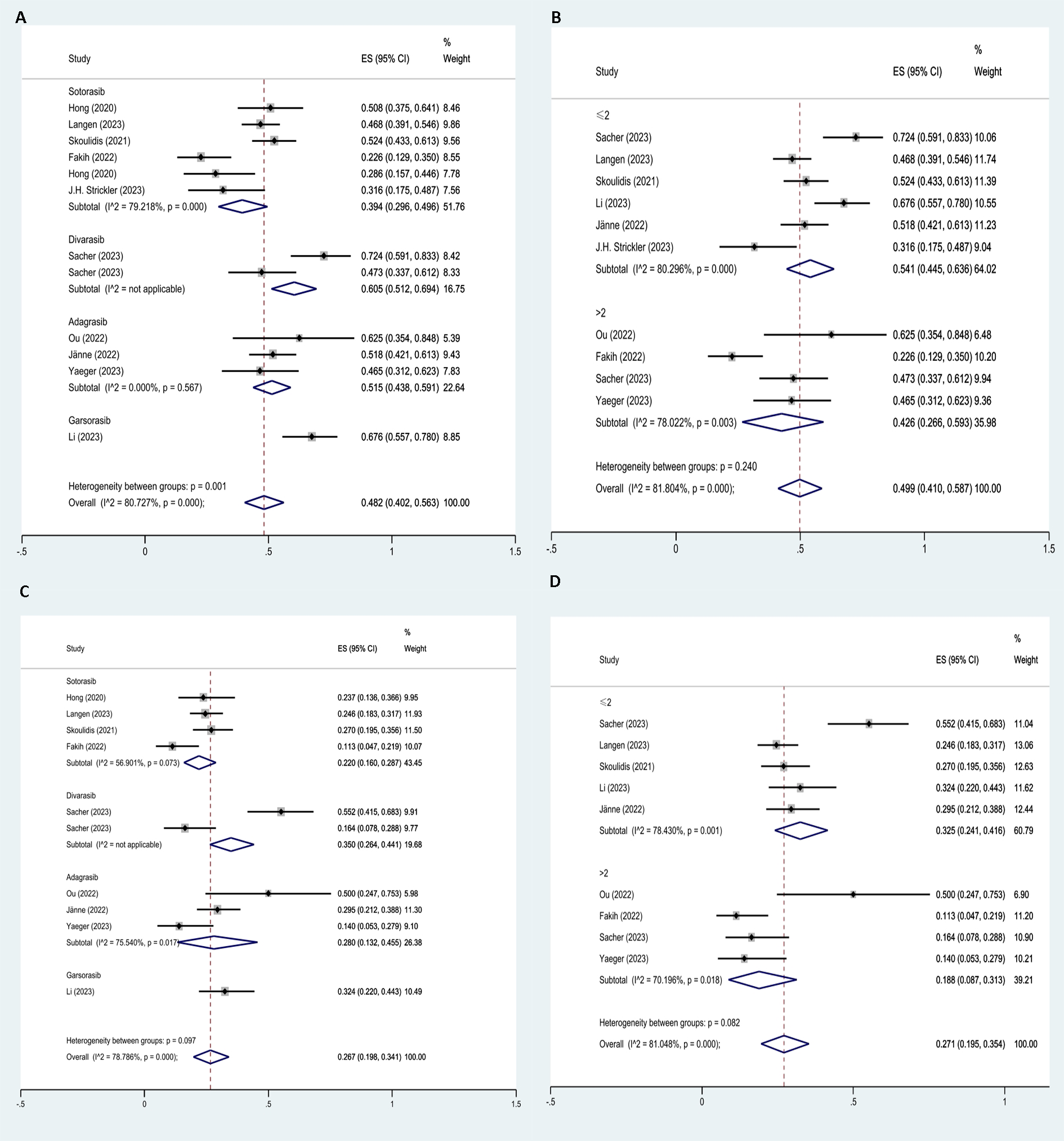


Figure S2. Subgroup analysis for PFS6 by drugs (A) and median NO. of previous treatment lines (B), subgroup analysis for PFS12 by drugs (C) and median NO. of previous treatment lines (D).


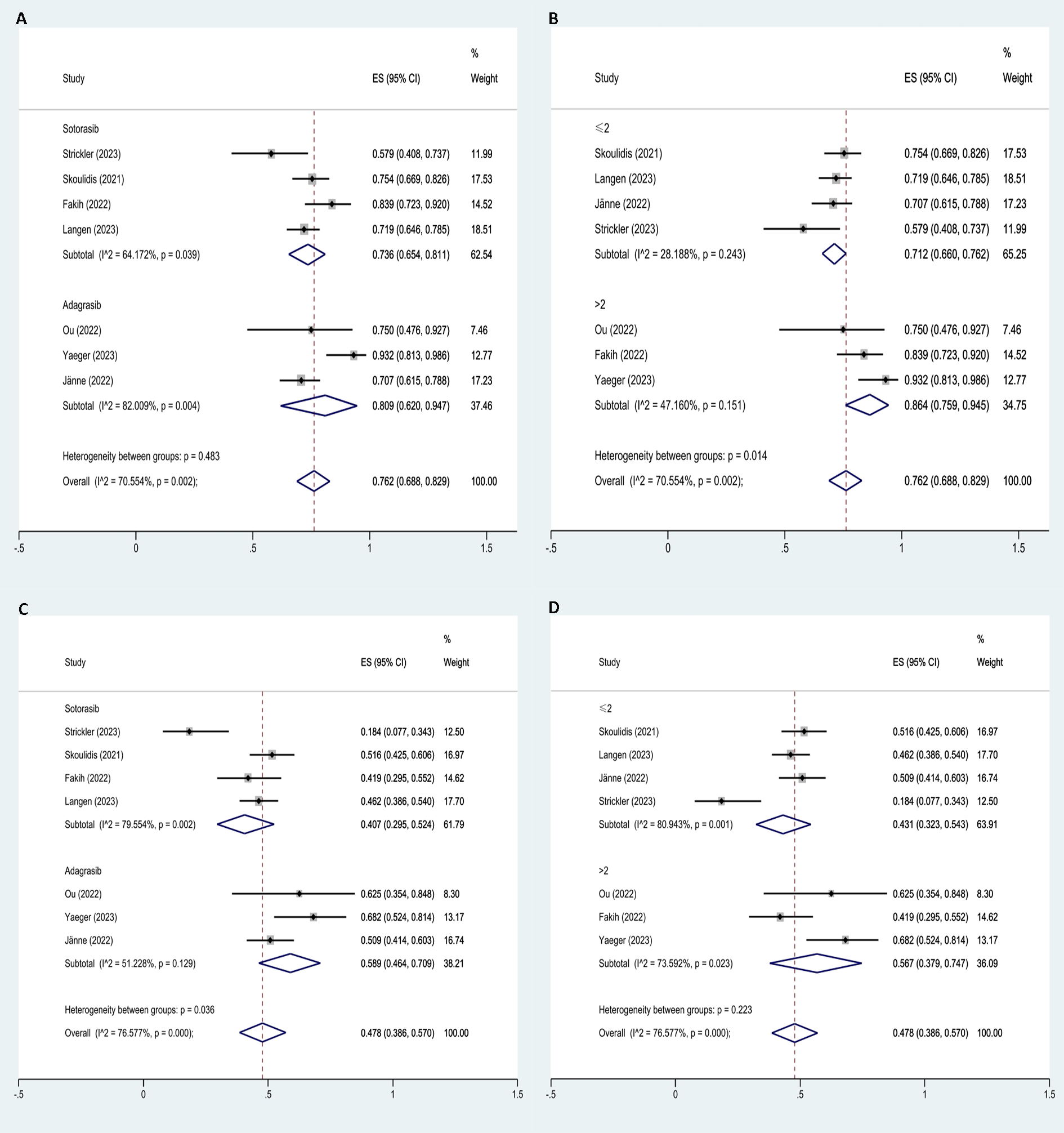


Figure S3. Subgroup analysis for OS6 by drugs (A) and median NO. of previous treatment lines (B), subgroup analysis for OS12 by drugs (C) and median NO. of previous treatment lines (D).


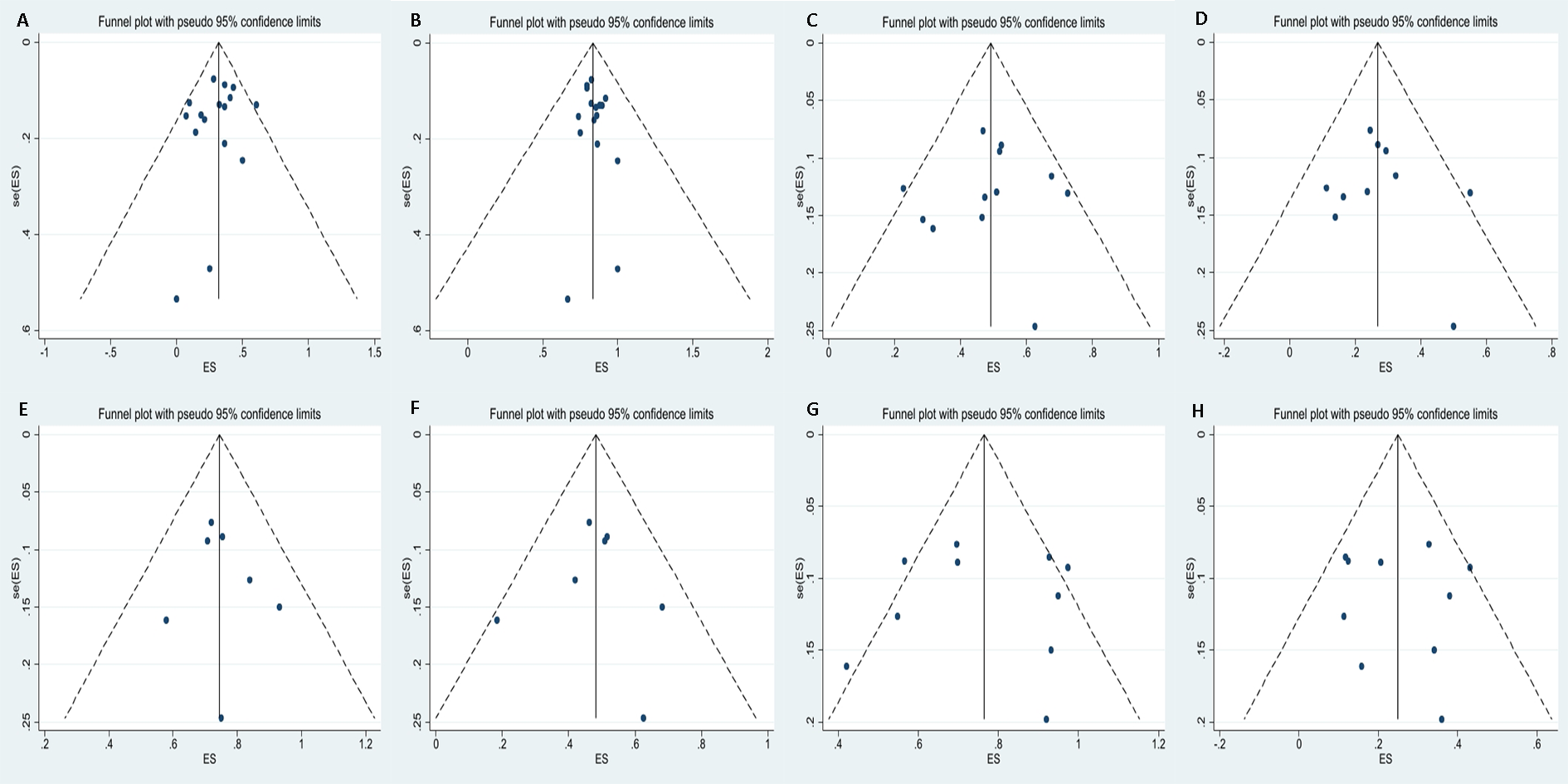


Figure S4. Publication bias analysis of ORR (A), DCR (B), PFS6 (C), PFS12 (D), OS6 (E), OS12 (F), incidence of any trAEs (G), and incidence of grade three or more teAEs (H).
